# Two consecutive myocardial tissue insults for inpatients with COVID-19

**DOI:** 10.1186/s13054-020-02981-9

**Published:** 2020-05-26

**Authors:** Minghua Su, Jieru Peng, Mengjun Wu, Wuquan Deng, Yousheng Yang, Yong G. Peng

**Affiliations:** 1 Department of Emergency, Sichuan Academy of Medical Sciences & Sichuan Provincial People’s Hospital, Chengdu, Sichuan People’s Republic of China; 2 Department of Medical Records Statistics, Chengdu Women and Children’ s Central Hospital, University of Electronic Science and Technology, Chengdu, Sichuan People’s Republic of China; 3 Department of Anesthesiology, Chengdu Women and Children’ s Central Hospital, School of medicine, University of Electronic Science and Technology of China, Chengdu, Sichuan People’s Republic of China; 4 Department of Endocrinology and Nephrology, Chongqing University Central Hospital & Chongqing Emergency Medical Center, No.1 Jiankang Road, Yuzhong District, Chongqing, 400014 People’s Republic of China; 5 Department of Intensive Care Unit, The Fifth Hospital of Wuhan, Wuhan, 610091 People’s Republic of China; 6 Department of Anesthesiology, University of Florida College of Medicine, Gainesville, FL 32610-0254 USA

The new coronavirus (COVID-19) originated in Wuhan, China, and has expeditiously spread across the global [1, 2]. Recent reports have suggested that myocardial injury was significantly associated with the fatality of the COVID-19 infection [3]. However, details of the risk factors leading to myocardial injury and death in adult inpatients with COVID-19 have not yet been well-described.

This was a retrospective case-control study (*n* = 105) with a confirmed novel coronavirus-infected pneumonia diagnosis between January 10, 2020, and February 20, 2020. It was approved by the institutional ethics board of The Fifth Hospital of Wuhan, Wuhan, P.R. China. Each individual non-survivor (*n* = 35) was matched with two subjects (*n* = 70) based on age, sex, and date of admission among discharged inpatients.

The non-survivors (*n* = 35) had a mean age of 64 years; 57% of them were male. Almost 70% of the non-survivors had comorbidities, including hypertension (51.4%), diabetes (14.3%), or others (34.3%). Concentrations of cardiac biomarkers in non-survivors were significantly higher than those of survivors. Compared with survivors, non-survivors had a statistically higher value of creatinine kinase-myocardial band (CK-MB; 15.64 ± 5.50 vs 5.39 ± 0.75; *p* < 0.001). The concentration of creatine kinase was dramatically increased in non-survivors (991.85 ± 368.71 vs 48.95 ± 5.51; *p* < 0.001). The mean consistence of lactate dehydrogenase in non-survivors was three times greater than that in survivors (653.94 ± 65.76 vs 281.70 ± 22.77; *p* = 0.008). The non-survivors had a statistically higher value of myoglobin (505.70 ± 91.52 vs 97.13 ± 23.87; *p* = 0.001) and Troponin I (10.72 ± 9.13 vs 0.08 ± 0.02; *p* = 0.002) in comparison with survivors. The serum urea nitrogen, creatinine, and high-sensitivity C-reactive protein (hs-CRP) in non-survivors was significantly elevated in comparison with survivors (16.54 ± 2.45 vs 2.87 ± 0.58, *p* < 0.001; 184.68 ± 41.27 vs 63.42 ± 6.21, *p* < 0.001; 217.47 ± 46.35 vs 27.65 ± 21.83, *p* = 0.002, respectively). Cardiac biomarkers in non-survivors had double-peak patterns: CK-MB peaked on the 3rd and 10th days after admission, myoglobin on the 3rd and 8th days, and Troponin I on the 5th and 8th days (Fig. 1).

**Fig. 1:**
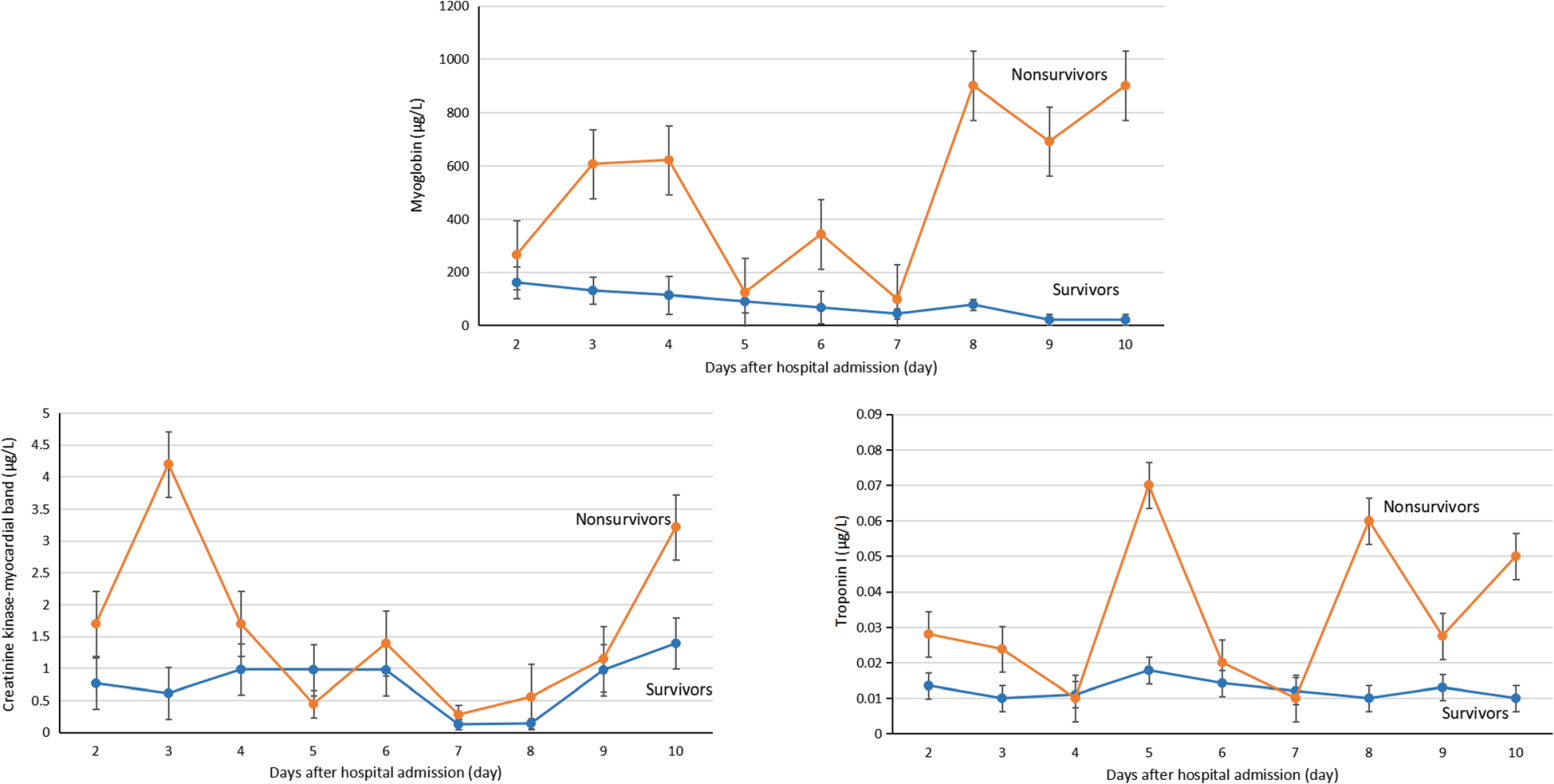
Timeline chart of creatinine kinase-myocardial band (CK-MB), myoglobin, and Troponin I concentration during hospitalization comparing survivors (blue line) with non-survivors (orange line)

Cardiac biomarkers (CK-MB, myoglobin, and Troponin I) in non-survivors had a double-peak phenomenon during hospitalization, which suggested that myocardial tissue may have suffered two consecutive insults.

At the early stage (first peak wave), the white cell count and neutrophil count were almost normal, and the lymphocyte counts decreased [4]. Meanwhile, infections were milder (serum hs-CRP level was low), and serum levels of myoglobin and CK-MB were inversely correlated with hs-CRP. However, at a later stage (second peak wave), the hs-CRP level continued to increase and peaked on the 7th day, which suggested a stronger inflammatory immune response, while these individuals also exhibited multiple organ dysfunction [4]. In addition, the association between myoglobin and CK-MB was moderately positive. Most importantly, in the latest pathological report, the degeneration and necrosis of cardiomyocytes for the non-survivors were confirmed to be caused by inflammatory destruction of cytokine storm [5]. From these results, myocardial injury might be associated with the direct insult of the virus at an early stage and a subsequent attack of the cytokine cascade (sustained inflammatory response) at a later stage.

## Data Availability

All data generated or analyzed during this study are included in this published article.
